# Iron Deficiency Impacts Diastolic Function, Aerobic Exercise Capacity, and Patient Phenotyping in Heart Failure With Preserved Ejection Fraction: A Subanalysis of the OptimEx-Clin Study

**DOI:** 10.3389/fphys.2021.757268

**Published:** 2022-02-10

**Authors:** Andreas B. Gevaert, Stephan Mueller, Ephraim B. Winzer, André Duvinage, Caroline M. Van de Heyning, Elisabeth Pieske-Kraigher, Paul J. Beckers, Frank Edelmann, Ulrik Wisløff, Burkert Pieske, Volker Adams, Martin Halle, Emeline M. Van Craenenbroeck, for the OptimEx-Clin Study Group

**Affiliations:** ^1^Research Group Cardiovascular Diseases, GENCOR (Genetics, Pharmacology & Physiopathology of Heart, Blood, Vessels and Skeleton) Department, University of Antwerp, Antwerp, Belgium; ^2^Department of Cardiology, Antwerp University Hospital (UZA), Edegem, Belgium; ^3^Department of Prevention and Sports Medicine, University Hospital Klinikum Rechts der Isar, Technical University of Munich, Munich, Germany; ^4^DZHK (German Center for Cardiovascular Research), Partner Site Munich Heart Alliance, Munich, Germany; ^5^Department of Internal Medicine and Cardiology, Heart Center Dresden – University Hospital, Technische Universität Dresden, Dresden, Germany; ^6^Department of Internal Medicine and Cardiology, Campus Virchow Klinikum, Charité Universitätsmedizin Berlin, Berlin, Germany; ^7^DZHK (German Center for Cardiovascular Research), Partner Site Berlin, Berlin, Germany; ^8^Cardiac Exercise Research Group at Department of Circulation and Medical Imaging, Norwegian University of Science and Technology, Trondheim, Norway

**Keywords:** heart failure, echocardiography, machine learning, exercise testing, diastolic dysfunction, iron deficiency, artificial intelligence, HFpEF

## Abstract

**Aims:**

Iron deficiency (ID) is linked to reduced aerobic exercise capacity and poor prognosis in patients with heart failure (HF) with reduced ejection fraction (HFrEF); however, data for HF with preserved ejection fraction (HFpEF) is scarce. We assessed the relationship between iron status and diastolic dysfunction as well as aerobic exercise capacity in HFpEF, and the contribution of iron status to patient phenotyping.

**Methods and Results:**

Among 180 patients with HFpEF (66% women; median age, 71 years) recruited for the Optimizing Exercise Training in Prevention and Treatment of Diastolic HF (OptimEx-Clin) trial, baseline iron status, including iron, ferritin, and transferrin saturation, was analyzed (*n* = 169) in addition to exercise capacity (peak oxygen uptake [peak V̇O_2_]) and diastolic function (E/e′). ID was present in 60% of patients and was more common in women. In multivariable linear regression models, we found that diastolic function and peak V̇O_2_ were independently related to iron parameters; however, these relationships were present only in patients with HFpEF and ID [E/e′ and iron: β−0.19 (95% confidence interval −0.32, −0.07), *p* = 0.003; E/e′ and transferrin saturation: β−0.16 (−0.28, −0.04), *p* = 0.011; peak V̇O_2_ and iron: β 3.76 (1.08, 6.44), *p* = 0.007; peak V̇O_2_ and transferrin saturation: β 3.58 (0.99, 6.16), *p* = 0.007]. Applying machine learning, patients were classified into three phenogroups. One phenogroup was predominantly characterized by the female sex and few HFpEF risk factors but a high prevalence of ID (86%, *p* < 0.001 vs. other phenogroups). When excluding ID from the phenotyping analysis, results were negatively influenced.

**Conclusion:**

Iron parameters are independently associated with impaired diastolic function and low aerobic capacity in patients with HFpEF and ID. Patient phenotyping in HFpEF is influenced by including ID.

**Clinical Trial Registration:**

www.ClinicalTrials.gov, identifier NCT02078947.

## Introduction

In patients with heart failure (HF) and reduced ejection fraction (HFrEF), iron deficiency (ID) is a known predictor of adverse outcomes (Jankowska et al., 2010; Klip et al., 2013). As iron is a key factor in erythropoiesis, energy metabolism, and mitochondrial function, disturbances in iron metabolism also impact functional status, including exercise capacity (Okonko et al., 2011). The diagnosis of ID in HFrEF is crucial, since intravenous correction of ID improves exercise performance, symptoms, and quality of life and might reduce hospitalizations, regardless of the presence of anemia (Anker et al., 2009; Ponikowski et al., 2015, 2020).

Less is known about the influence of iron status on functional status and myocardial performance in HF with preserved ejection fraction (HFpEF). HFpEF accounts for half of the HF hospital admissions, and its pathophysiology is characterized by both cardiac (e.g., diastolic dysfunction) and non-cardiac (e.g., skeletal myopathy) abnormalities causing exercise intolerance (Shimiaie et al., 2015; Gevaert et al., 2019; Pugliese et al., 2019). Observational data show a strong relationship between ID and a reduced aerobic exercise capacity, measured as peak oxygen uptake (peak V̇O_2_), in patients with HFpEF (Beale et al., 2019). In addition, ID affects immune responses and oxidative stress mechanisms underlying these cardiac and non-cardiac abnormalities (Macdougall et al., 2012; Paulus and Tschöpe, 2013). *In vitro*, ID is associated with impaired mitochondrial respiration and reduced contractility and relaxation in human cardiomyocytes (Hoes et al., 2018). However, a relationship of ID to either exercise capacity or left ventricular (LV) stiffness could not be found in a small study of patients with HFpEF (Kasner et al., 2013).

While several drugs are able to reduce HF hospitalizations, a therapy that improves mortality in HFpEF is still lacking (Gevaert et al., 2022). A key reason for this could be the underlying phenotypic heterogeneity of HFpEF (Shah et al., 2016). Subdividing and classifying patients with HFpEF by different disease phenotypes may increase our understanding of HFpEF pathophysiology, simplify its diagnosis, and allow for targeted management instead of a “one size fits all” approach (Shah et al., 2016; Gevaert et al., 2020).

Artificial intelligence can aid in this classification by recognizing patterns in large datasets. In unsupervised machine learning, relationships are determined based on raw data, independent of the existing classification. One approach is to find “clusters” of similar data items: subjects in the same cluster are similar to each other and dissimilar to subjects in other clusters. Previously, machine learning has been able to identify clusters of patients with different prognoses in patients with HFrEF and/or HFpEF (Kao et al., 2015; Shah et al., 2015; Hedman et al., 2020).

In this study, we assessed the iron status of patients with HFpEF included in the Optimizing Exercise Training in Prevention and Treatment of Diastolic Heart Failure (OptimEx-Clin) multicenter trial (Mueller et al., 2021). We aimed to establish the relationship of iron parameters with diastolic function and aerobic exercise capacity, and the contribution of iron status to patient phenotyping using machine learning.

## Materials and Methods

### Patients and Study Design

This is a subanalysis of the baseline data of the OptimEx-Clin study, whose rationale, design, and primary outcomes have been published previously (Suchy et al., 2014; Mueller et al., 2021). Briefly, OptimEx-Clin was a randomized multicenter trial assessing the effect of different training modalities on peak V̇O_2_ in patients with HFpEF. This study was conducted between September 2014 and July 2018 at the Technical University of Munich, Heart Center Leipzig, and Charité Universitätsmedizin Berlin in Germany, and Antwerp University Hospital in Belgium. Inclusion criteria were (i) signs and symptoms of HF using the New York Heart Association classes II or III, (ii) LV ejection fraction ≥ 50%, (iii) diastolic dysfunction defined as E/e′ > 15 or E/e′ > 8 + *N*-terminal pro-B-type natriuretic peptide (NT-proBNP) > 220 pg/mL (Paulus et al., 2007), (iv) sedentary lifestyle, and (v) optimal medical treatment and clinically stable for ≥ 6 weeks.

This study complied with the Declaration of Helsinki and was approved by local ethics committees at the participating centers. We obtained written informed consent from each participant.

### Laboratory Analysis and Definitions

Iron status (i.e., serum iron, ferritin, and total iron-binding capacity) was assayed centrally at the Antwerp University Hospital using a Dimension Vista 1,500 system (Siemens) and could be determined in 169 of 180 patients randomized to intervention. Hemoglobin (Hb) was assayed locally. NT-proBNP was measured centrally at the Clinical Institute of Medical and Chemical Laboratory Diagnostics, Medical University of Graz, Austria. Transferrin saturation was calculated as serum iron/total iron-binding capacity. Absolute ID was defined as ferritin < 100 μg/L. Functional ID was defined as ferritin 100–299 μg/L with transferrin saturation < 20% (Anker et al., 2009). Anemia was defined as Hb < 120 g/L in women and Hb < 130 g/L in men.

### Echocardiography and Cardiopulmonary Exercise Testing

Echocardiography data were analyzed and blinded at the echocardiography core lab (Berlin, Germany). Cardiopulmonary exercise testing (CPET) data were analyzed and blinded at the CPET core lab (Munich, Germany). Protocols were described previously (Suchy et al., 2014; Mueller et al., 2021). Lean body mass was calculated as total weight—(1/body fat), with body fat calculated using the Gallagher formula (Gallagher et al., 2000). Predicted peak heart rate was calculated according to the study by Nes et al. (2013).

### Unsupervised Machine Learning

For machine learning and statistical analysis, we used R software (R Foundation for Statistical Computing) version 3.5.1. All 176 patients who met the inclusion criteria for OptimEx-Clin were included for this analysis. First, we evaluated all 94 baseline clinical, echocardiographic, and CPET variables for redundancy (Supplementary Table 1). We filtered variables that had > 90% identical values, > 30% missing values, or correlated at Spearman’s correlation coefficient of > 0.5, keeping the variable that was most informative and had the least missingness. In addition, variables that were used in the calculation of another variable were filtered regardless of their correlation coefficient [e.g., weight and height were filtered, and body mass index (BMI) was retained] (Shah et al., 2015; Gevaert et al., 2021). Thus, we retained 33 of 94 variables (Supplementary Table 1).

Next, a dissimilarity matrix was computed using the Gower distance, which was chosen because it can handle categorical variables and missing values (Kaufman and Rousseeuw, 1990). For missing values, two cases were compared if there was at least one variable where both had correct values. The optimal algorithm for clustering was assessed by comparing multiple internal validation and cluster stability statistics and 1,000-fold bootstrap resampling (*clValid* and *fpc* packages) (Hennig, 2007). The optimal number of clusters was assessed using the gap statistics (*cluster* package) (Tibshirani et al., 2001). Agglomerative hierarchical clustering with Ward linkage was superior to other strategies. We then repeated the analysis excluding ID.

### Statistical Analysis

Baseline characteristics were compared between patients with ID vs. without ID, and between clusters found by unsupervised machine learning. In normally distributed data (the Shapiro–Wilk test) with equal variances (Levene’s test), we used a one-way analysis of variance (ANOVA). In case of unequal variances, we used a White-corrected one-way ANOVA instead *(car* package). In non-normally distributed variables, we used the Kruskal-Wallis test instead. For categorical variables, Pearson’s chi-square test was used.

A logistic regression model was constructed for ID and sex, and the odds ratio with 95% confidence interval (CI) was computed. E/e′ septal ratio and peak V̇O_2_ were defined as parameters of interest for linear models. For univariate analysis, Pearson’s correlation coefficients were used. A multivariable linear regression model was constructed for each significant correlation between iron status (e.g., iron, ferritin, and transferrin saturation) vs. the parameters of interest. Age and sex were predefined as covariates and were added in one block to the model. The population was then stratified by iron status, and regressions were repeated.

Model diagnostics included residual distributions, standardized residuals vs. fitted values plots, and quantile-quantile plots (*gvlma* package). If anomalies were noted on diagnostics, analysis was repeated after logarithmic transformation of the outcome variable. A two-sided *p*-value < 0.05 was considered significant. *P*-values were adjusted for multiple comparisons according to the Holm method or Dunn method after the Kruskal-Wallis test (*multcomp* package).

## Results

### Patient characteristics

In OptimEx-Clin, 180 patients were randomized, of whom 4 were excluded after blinded review of eligibility (Mueller et al., 2021). Clinical characteristics of 169 patients with available iron measurements are shown in Table 1, stratified according to iron status. For the total population, patients were elderly [median age, 71 (65–76) years] and predominantly women (66%). Typical comorbidities included hypertension (85%), dyslipidemia (71%), obesity (44%), chronic kidney disease (39%), coronary artery disease (CAD) (27%), and atrial fibrillation (27%). Patients were treated with angiotensin-converting enzyme inhibitors or angiotensin receptor blockers (71%), beta-blockers (64%), diuretics (54%), and statins (53%). Patients were well-compensated with a median NT-proBNP value of 299 (146–620) pg/mL and 74% of patients were grouped in New York Heart Association class II.

**TABLE 1:** Characteristics of the study population, stratified according to the presence of iron deficiency.

Characteristic	Iron deficient (*n* = 101)		Normal iron status (*n* = 68)		*P*-value
Age (years)	69	(64–76)	72	(68–76)	0.091
Sex (n,% female)	77	(76)	34	(50)	< 0.001
**Medical history**					
Atrial fibrillation	31	(31)	18	(26)	0.674
Cerebrovascular disease	16	(16)	4	(6)	0.085
Chronic kidney disease	35	(35)	26	(38)	0.755
Chronic obstructive pulmonary disease	5	(5)	7	(10)	0.307
Coronary heart disease	27	(28)	23	(34)	0.529
Diabetes	27	(27)	18	(27)	1.000
Family history of cardiovascular disease	27	(27)	12	(18)	0.254
Hypertension	84	(83)	63	(93)	0.118
Hyperlipidemia	75	(76)	44	(65)	0.169
Peripheral vascular disease	7	(7)	2	(3)	0.433
Sleep apnea	15	(15)	17	(26)	0.137
Smoking, current or previous	47	(47)	29	(43)	0.733
Valvular heart disease	6	(6)	3	(4)	0.932
**Medication use**					
ACE inhibitor or ARB	66	(65)	60	(88)	0.002
Aldosterone antagonist	9	(9)	10	(15)	0.357
Anticoagulant	37	(37)	19	(28)	0.312
Antiplatelet	34	(34)	25	(37)	0.802
Beta-blocker	63	(62)	49	(72)	0.254
Calcium antagonist	28	(28)	34	(50)	0.005
Diuretic	51	(50)	47	(69)	0.025
Glucose lowering	24	(24)	15	(22)	0.943
Lipid lowering	54	(53)	50	(59)	0.596
**Clinical examination**					
Blood pressure, systolic (mmHg)	128	± 14	128	± 14	0.951
Blood pressure, diastolic (mmHg)	75	± 10	74	±10	0.460
Body mass index (kg/m^2^)	29.1	(25.7–32.1)	29.9	(27.1–34.8)	0.060
KCCQ symptom score	70	(52–81)	75	(56–82)	0.449
NYHA class					
II (n,%)	76	(75)	50	(74)	0.943
III (n,%)	25	(25)	18	(26)	
Rest heart rate (bpm)	65	± 11	64	±9	0.490
**Laboratory analysis**					
Iron (μmol/L)	15.2	(11.8–18.8)	17.5	(14.6–21.1)	0.003
Ferritin (μg/L)	49	(34–79)	476	(318–705)	< 0.001
Transferrin saturation (%)	23.2	(17.6–29.5)	28.3	(24.0–35.2)	< 0.001
Anemia* (n,%)	25	(25)	11	(16)	0.253
Hemoglobin (g/L)	133	± 15	138	± 14	0.030
EGFR (mL/min/1.73 m^2^)	70.8	(58.0–86.7)	71.9	(56.1–83.6)	0.831
NT-proBNP (pg/mL)	317	(150–604)	285	(122–629)	0.839
**Cardiopulmonary exercise test**					
Peak heart rate (bpm)	124	± 27	120	± 24	0.313
Percent predicted peak heart rate (%)	74	± 16	72	±14	0.464
Peak V̇O_2_ (mL/kg/min)	18.6	± 5.7	19.2	± 4.8	0.476
Peak V̇O_2_ per lean body mass (mL/kg/min)	29.9	± 8.8	30.6	± 7.2	0.592
Percent predicted peak V̇O_2_ (%)	80.9	(66.7–99.1)	89.4	(70.2–105.9)	0.083
V̇O_2_ at the aerobic threshold (mL/min)	857	± 208	1,025	± 298	< 0.001
Peak VO_2_ pulse (mL/beat)	11.8	± 3.3	14.2	± 3.6	< 0.001
Peak workload (W)	93	(72–117)	100	(84–119)	0.093
V̇E/V̇CO_2_ slope	33.9	(29.4–39.3)	31.3	(28.5–35.4)	0.015
**Echocardiography**					
E/A ratio	1.08	(0.85–1.40)	0.93	(0.79–1.20)	0.033
E/e’ ratio, septal	14.8	(13.1–17.0)	15.6	(12.5–18.1)	0.597
Left atrial volume index (mL/m^2^)	34.7	(29.5–45.0)	34.7	(29.3–44.3)	0.540
LV mass index (g/m^2^)	155	(132–203)	190	(166–230)	0.004
LV ejection fraction (%)	60	(55–64)	61	(55–64)	0.512
PAPs (mmHg)	30.4	± 8.2	31.5	± 7.6	0.403
TAPSE (mm)	21.3	± 3.5	21.7	± 3.7	0.518

Continuous variables with normal distribution: mean ± SD and t-test; continuous variables with skewed distribution: median (25th–75th percentile) and Wilcoxon’s rank sum test; categorical variables: n (% of total) and chi-square test. *Anemia was defined as hemoglobin < 120 g/L in women and < 130 g/L in men.

*ACE, angiotensin-converting enzyme, ARB, angiotensin receptor blocker; EGFR, estimated glomerular filtration rate (CKD-EPI formula); KCCQ, Kansas City Cardiomyopathy Questionnaire; LV, left ventricular; NT-proBNP, N-terminal-pro-B-type natriuretic peptide; NYHA, New York Heart Association; PAPs, systolic pulmonary artery pressure; TAPSE, tricuspid annular plane systolic excursion; V̇E, ventilation; V̇CO_2_, carbon dioxide production; V̇O_2_, oxygen uptake.*

### Iron Status in Patients With HF With Preserved Ejection Fraction

ID was present in 101 (60%) patients at baseline. Of patients with ID, 35 (34%) had transferrin saturation < 20% and 95 (94%) had ferritin < 100 mg/dl. Women were more prone to have ID (women 69%, men 41%, odds ratio 3.21, CI 1.67–6.28, *p* = 0.0005). Patients with ID had lower iron, ferritin, transferrin saturation, and Hb (all *p* < 0.05, Table 1). Anemia was present in 36 (21%) patients, of whom 25 (69%) had concurrent ID.

There were no differences in the medical history or clinical parameters between patients with and without ID (Table 1). Patients without ID more frequently took angiotensin blockers, calcium antagonists, and diuretics. Patterns of antiplatelet or anticoagulant drug use did not differ between patients with and without ID (Table 1).

The CPET demonstrated a similar peak V̇O_2_ in patients with or without ID; however, other parameters associated with the prognosis of patients with HF were significantly worse in patients with ID, including steeper ventilation/carbon dioxide production (VE/VCO_2_) slope, lower peak O_2_ pulse, and lower V̇O_2_ at aerobic threshold (Table 1). Echocardiography revealed a higher E/A ratio, and lower LV mass, in patients with ID (Table 1).

### Association With Cardiac Diastolic Function and Aerobic Exercise Capacity

Patients with lower transferrin saturation or lower serum iron had a higher E/e′ ratio (Table 2). There were no significant correlations between iron status and systolic pulmonary artery pressure, left atrial volume, or LV ejection fraction. Patients with lower transferrin saturation or lower serum iron also had a lower peak V̇O_2_ (Table 2). There were no significant correlations of ferritin with either diastolic function or aerobic exercise capacity.

**TABLE 2:** Associations of baseline iron status with cardiac function and aerobic exercise capacity.

	Pearson correlation		Multivariable linear regression			
	r	*p*	β	95% CI	Adj. *R*^2^	*p*
**All patients (*n* = 169)**						
**E/e’ septal (ratio)**						
Iron (μmol/L)	–0.163	**0.035**	–0.129	–0.244, –0.014	0.031	**0.029**
Transferrin saturation (%)	–0.155	**0.045**	–0.113	–0.221, –0.006	0.027	**0.039**
**Peak V̇O_2_ (mL/min/kg)**						
Iron (μmol/L)	0.186	**0.015**	2.629	0.526, 4.731	0.145	**0.015**
Transferrin saturation (%)	0.196	**0.011**	2.370	0.418, 4.323	0.144	**0.018**
**Iron deficiency (*n* = 101)**						
**E/e’ septal (ratio)**						
Iron (μmol/L)	–0.253	**0.012**	–0.193	–0.318, –0.068	0.086	**0.003**
Transferrin saturation (%)	–0.232	**0.022**	–0.161	–0.284, –0.039	0.064	**0.011**
**Peak V̇O_2_ (mL/min/kg)**						
Iron (μmol/L)	0.269	**0.006**	3.758	1.075, 6.442	0.164	**0.007**
Transferrin saturation (%)	0.301	**0.002**	3.578	0.993, 6.163	0.164	**0.007**
**No iron deficiency (*n* = 68)**						
**E/e’ septal (ratio)**						
Iron (μmol/L)	–0.021	0.866	0.005	–0.255, 0.265	–0.031	0.970
Transferrin saturation (%)	–0.030	0.809	–0.040	–0.283, 0.203	–0.030	0.742
**Peak V̇O_2_ (mL/min/kg)**						
Iron (μmol/L)	–0.036	0.769	–0.419	–4.267, 3.428	0.111	0.828
Transferrin saturation (%)	0.067	0.586	–0.681	–4.272, 2.910	0.112	0.706

*Multivariable linear regressions were adjusted for age and sex. All variables expect peak V̇O_2_ were log transformed before analysis. Adj, adjusted. Bold type: p < 0.05.*

In multivariable linear regression models, the relationships of lower iron parameters (e.g., iron, transferrin saturation) with reduced E/e′ ratio and lower peak V̇O_2_ were confirmed to be independent of age and sex (Table 2). However, the proportion of variance in diastolic function or peak V̇O_2_ explained by iron status was small (all adjusted *R*^2^ < 0.2).

**FIGURE 1:**
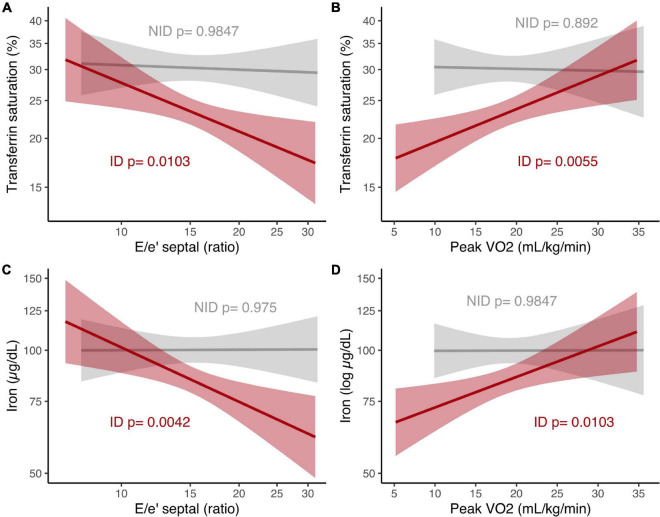
Relationship of iron parameters to E/e′ septal ratio and peak V̇O_2_ in patients with HFpEF, stratified according to iron status. **(A)** Transferrin saturation and E/e′ septal ratio, both log scale. **(B)** Transferrin saturation (log scale) and peak V̇O_2_. **(C)** Iron and E/e′ septal ratio, both log scale. **(D)** Iron (log scale) and peak V̇O_2_. *P*-values from linear regression analyses in patients with ID and without ID. ID, iron deficiency; NID, no iron deficiency; peak V̇O_2_, peak oxygen uptake.

We then stratified patients according to the presence of ID and repeated the regression analyses. This demonstrated that the relationship between iron parameters and diastolic function or peak V̇O_2_ only exists in patients with ID (Figure 1 and Table 2). In patients without ID, there was no significant correlation between iron parameters on the one hand, and diastolic function or aerobic exercise capacity on the other hand (Figure 1). These results were altered only marginally when including Hb as a covariate (data not shown).

### Iron Deficiency and HF With Preserved Ejection Fraction Phenotypes

**FIGURE 2:**
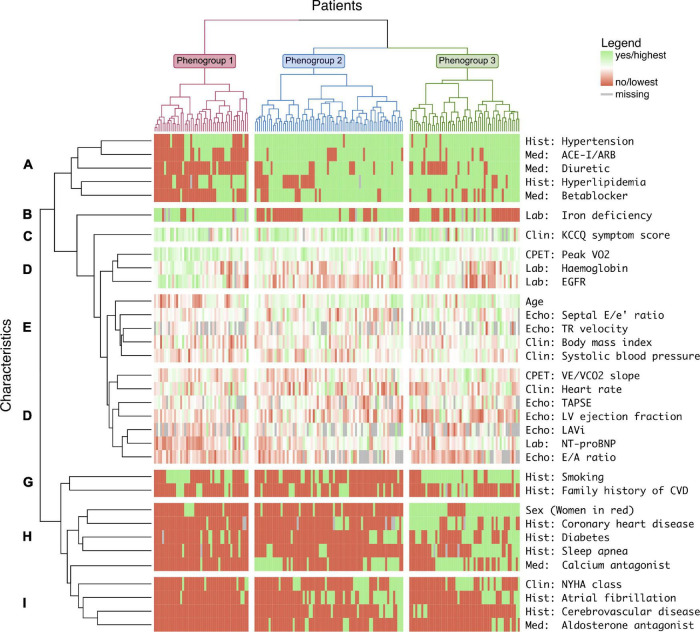
Three phenogroups of patients with HFpEF identified through machine learning and their characteristics. Heatmap with columns representing individual patients and rows representing individual characteristics, both grouped in clusters by unsupervised machine learning. Three phenogroups (numbers 1–3) and 9 characteristics clusters (letters A–I) were distinguished. ACE, angiotensin-converting enzyme; ARB, angiotensin receptor blocker; Clin, clinical examination; CVD, cardiovascular disease; Echo, echocardiography; EGFR, estimated glomerular filtration rate; Hist, medical history; KCCQ, Kansas City Cardiomyopathy Questionnaire; LAVi, left atrial volume index; LV, left ventricular; Med, current medication; NT-proBNP, *N*-terminal pro-B-type natriuretic peptide; NYHA, New York Heart Association; TAPSE, tricuspid annular plane systolic excursion; TR, tricuspid regurgitation; V̇CO_2_, carbon dioxide production; V̇E, ventilation; V̇O_2_, oxygen uptake.

Unsupervised clustering using relevant baseline variables from all 176 patients included in OptimEx-Clin resulted in three distinct phenogroups (i.e., bootstrap Jaccard coefficients 0.68, 0.66, and 0.66; > 0.6 indicating stable clusters; gap statistics for 3 clusters = 0.378 ± 0.012, indicating 3 clusters as optimum; Supplementary Figure 1). Figure 2 and Table 3 show baseline variables stratified per phenogroup.

**TABLE 3:** Characteristics of the study population, stratified according to phenogroup.

Characteristic	Phenogroup 1 (*n* = 47)		Phenogroup 2 (*n* = 74)		Phenogroup 3 (*n* = 55)		*P*-value
Age (years)	65	(56–69)*^†^	72	(68–76)	74	(69–77)	< 0.001
Sex (n,% female)	41	(77)^†^	67	(91)^†^	9	(16)	< 0.001
**Medical history**							
Atrial fibrillation	3	(6)*^†^	31	(42)	15	(27)	< 0.001
Cerebrovascular disease	0	(0)*	13	(18)	7	(13)	0.010
Chronic kidney disease	11	(23)	32	(43)	21	(38)	0.090
Chronic obstructive pulmonary disease	0	(0)	5	(7)	8	(15)	0.012
Coronary heart disease	2	(4)^†^	10	(14)^†^	38	(70)	< 0.001
Diabetes	7	(15)^†^	13	(18)^†^	26	(47)	< 0.001
Family history of cardiovascular disease	10	(21)	16	(21)	13	(25)	0.917
Hypertension	25	(53)*^†^	73	(99)	52	(95)	< 0.001
Hyperlipidemia	23	(50)*^†^	51	(70)^†^	49	(89)	< 0.001
Peripheral vascular disease	1	(2)	1	(1)	7	(13)	0.008
Sleep apnea	4	(9)^†^	7	(9)^†^	22	(42)	< 0.001
Smoking, current or previous	22	(47)*^†^	14	(19)^†^	43	(78)	< 0.001
Valvular heart disease	2	(4)	3	(4)	4	(7)	0.677
**Medication use**							
ACE inhibitor or ARB	14	(30)*^†^	66	(89)	49	(89)	< 0.001
Aldosterone antagonist	0	(0)*	12	(16)	7	(13)	0.013
Anticoagulant	5	(11)*	34	(46)	17	(31)	0.001
Antiplatelet	9	(19)^†^	14	(19)^†^	38	(69)	< 0.001
Beta-blocker	11	(23)*^†^	62	(84)	41	(75)	< 0.001
Calcium antagonist	2	(4)*^†^	33	(45)	28	(51)	< 0.001
Diuretic	5	(11)*^†^	60	(81)	37	(67)	< 0.001
Glucose lowering	4	(9)^†^	10	(14)^†^	16	(29)	0.011
Lipid lowering	9	(19)*^†^	40	(54)^†^	47	(85)	< 0.001
**Clinical examination**							
Blood pressure, systolic (mmHg)	125	± 14	126	± 15	131	± 12	0.114
Blood pressure, diastolic (mmHg)	77	± 9	74	±11	73	± 10	0.072
Body mass index (kg/m^2^)	28.2	(24.4–31.3)	30.3	(27.0–33.5)	29.6	(27.0–33.2)	0.072
KCCQ symptom score	68	(46–81)	71	(54–80)	74	(55–84)	0.810
NYHA class							
II (n,%)	42	(89)	52	(70)	36	(65)	0.013
III (n,%)	5	(11)^†^	22	(30)	19	(35)	
Rest heart rate (bpm)	67	± 10	63	±11	64	± 9	0.163
**Laboratory analysis**							
Iron deficiency (n,%)	37	(86)*^†^	42	(58)	22	(42)	<0.001
Iron (μmol/L)	15.0	(12.1–19.8)	16.7	(13.3–20.8)	16.6	(13.6–19.7)	0.506
Ferritin (μg/L)	66	(35–93)*^†^	91	(48–207)	141	(43–219)	0.002
Transferrin saturation (%)	24.9	(18.9–31.8)	25.8	(21.5–32.4)	24.7	(21.5–31.3)	0.660
Anemia (n,%)	7	(16)	13	(18)	16	(30)	0.149
Hemoglobin (g/L)	136	± 15	133	± 14	136	± 16	0.497
EGFR (mL/min/1.73 m^2^)	85.6	(71.8–94.8)*^†^	67.8	(54.9–76.9)	69.0	(53.5–82.6)	< 0.001
NT-proBNP (pg/mL)	153	(56–248)*^†^	379	(189–736)	332	(191–620)	< 0.001
**Cardiopulmonary exercise test**							
Peak heart rate (bpm)	141	± 24*^†^	116	± 24	117	± 24	< 0.001
Percent predicted peak heart rate (%)	83	± 13*^†⁣†^	69	±14	71	± 14	< 0.001
Peak V̇O_2_ (mL/kg/min)	21.0	± 5.7*	17.6	± 4.9	18.7	± 5.0	0.002
Peak V̇O_2_ per lean body mass (mL/kg/min)	34.0	± 7.2*^†^	29.8	± 8.1	27.8	± 6.6	< 0.001
Percent predicted peak V̇O_2_ (%)	82.3	(70.0–98.5)	81.7	(66.9–96.6)	87.7	(70.0–113.4)	0.143
V̇O_2_ at aerobic threshold (mL/min)	918	± 299	846	± 208^†^	1,007	± 260	0.002
Peak VO_2_ pulse (mL/beat)	11.2	± 2.9^†^	12.2	± 3.5^†^	14.4	± 3.6	< 0.001
Peak workload (W)	107	(88–134)*	90	(69–107)^†^	105	(81–124)	0.002
V̇E/V̇CO_2_ slope	31.8	(28.7–36.0)	32.3	(28.6–35.5)^†^	34.5	(30.4–42.0)	0.028
**Echocardiography**							
E/A ratio	1.05	(0.83–1.22)	1.18	(0.84–1.58)	0.97	(0.79–1.16)	0.099
E/e’ ratio, septal	14.6	(13.7–16.1)	15.0	(11.9–17.5)	16.0	(13.5–18.5)	0.098
Left atrial volume index (mL/m^2^)	32.3	(27.4–36.2)*	40.1	(32.3–46.6)^†^	33.8	(27.1–40.5)	0.003
LV mass index (g/m^2^)	155	± 43*^†^	178	± 40^†^	217	± 55	< 0.001
LV ejection fraction (%)	60	(58–63)	61	(56–64)	58	(55–65)	0.283
PAPs (mmHg)	28.3	(25.4–31.4)	30.3	(24.6–35.1)	31.0	(27.9–35.0)	0.145
TAPSE (mm)	22.1	± 3.4	20.7	± 3.7	22.0	± 3.7	0.076

*Normally distributed variables: mean ± SD and one-way ANOVA. Skewed variables: median (interquartile range) and Kruskal-Wallis test. Categorical variables: n (%) and Pearson’s chi-square test.*

*ACE, angiotensin-converting enzyme; ARB, angiotensin receptor blocker; EGFR, estimated glomerular filtration rate; LV, left ventricular; NT-proBNP, N-terminal pro-B-type natriuretic peptide; NYHA, New York Heart Association; PAPs, systolic pulmonary artery pressure; TAPSE, tricuspid annular plane systolic excursion; V̇CO_2_, carbon dioxide production; V̇E, ventilation; V̇O_2_, oxygen uptake.*

*Multiple comparisons-adjusted p < 0.05 vs. phenogroup 2. ^†^Multiple comparisons-adjusted p < 0.05 vs. phenogroup 3.

In *phenogroup 1* (*n* = 47), patients were the youngest (median age, 65 years) and predominantly women (77%), had fewer comorbidities, and were least likely to use cardiovascular drugs (Figures 2A,E,H). Their estimated glomerular filtration rate was the highest, NT-proBNP levels were the lowest, and CPET performance was the best (Figures 2D,F). In contrast, the prevalence of ID was remarkably high in this phenogroup (86%, Figure 2B).

In *phenogroup 2* (*n* = 74), patients aged 72 years on average and were predominantly women (91%). Cardiovascular risk factors were highly prevalent, including hypertension (99%) and hyperlipidemia (70%). This phenogroup had the highest prevalence of atrial fibrillation (42%, Figure 2I) and chronic kidney disease (43%).

In *phenogroup 3* (*n* = 55), patients were predominantly men (84%). Again, patients were elderly (median age, 74 years) and > 90% had hypertension and hyperlipidemia. This phenogroup has a remarkably high prevalence of coronary heart disease (70%), clustering with diabetes, male sex, and sleep apnea (Figure 2H). Patients in phenogroup 3 had a higher prevalence of diabetes (47%).

Repeating the clustering analysis without including ID as a variable resulted in 8 phenogroups that were significantly less stable (patients more prone to be allocated to another cluster when repeating the analysis) with a mean bootstrap Jaccard coefficient of 0.48 ± 0.13, compared to 0.67 ± 0.01 in the initial analysis (*p* = 0.005) (Supplementary Figures 2, 3 and Supplementary Table 2).

## Discussion

We concluded that in patients with HFpEF, (i) ID is a very frequent comorbidity, (ii) iron parameters relate to diastolic function and aerobic exercise capacity only in patients with ID, (iii) patient phenotyping is significantly impacted by including ID, and (iv) a phenogroup of younger women with HFpEF has a high prevalence of ID but a few other comorbidities. Together, this forms evidence of a clinically important link between HFpEF and ID in some but not all patients.

The guideline-recommended management of HFpEF is still based on treating symptoms and controlling comorbidities (Yancy et al., 2017). In this study, we confirmed that ID is an important comorbidity in HFpEF. Affecting 59% of this multicenter European HFpEF population, ID is more prevalent than obesity, diabetes mellitus, CAD, chronic kidney disease, or atrial fibrillation. Previous reports in smaller HFpEF populations found a comparable prevalence of ID, ranging from 57 to 70% (Kasner et al., 2013; Núñez et al., 2016; Martens et al., 2018). ID seems more common in HFpEF than HFrEF, where a prevalence of 37–50% is reported (Jankowska et al., 2010; Okonko et al., 2011; Klip et al., 2013; Grote Beverborg et al., 2018). This could be explained by the larger proportion of women among patients with HFpEF, as female sex is also a known risk factor for ID (Beale et al., 2018).

Indeed, in our study, women had a threefold higher risk of ID compared to men. The cause of this sex difference is not clear, as ID in women is traditionally attributed to blood loss during menstruation and higher iron requirements during pregnancy (Beale et al., 2018), but all women in our study were postmenopausal. Other risk factors shared by HFpEF and ID (e.g., age, chronic kidney disease, and obesity) did not differ between patients with and without ID in our study.

In multivariable regression, the relationship between iron parameters and peak V̇O_2_ was minor. This confirms findings from a recent analysis in 300 patients with HFpEF (Barandiarán Aizpurua et al., 2021). Previously, smaller studies found more important relationships between iron parameters and peak V̇O_2_ (Núñez et al., 2016; Martens et al., 2018). Patients in these studies had a much lower exercise capacity than the current population, so disease severity is possibly a factor to consider in the relationship between iron status and aerobic exercise capacity. However, several other CPET parameters showed unfavorable changes in patients with ID: steeper VE/VCO_2_ slope, lower peak O_2_ pulse, and lower V̇O_2_ at aerobic threshold (Table 1). These parameters have all been associated with the prognosis of patients with HF (Guazzi et al., 2012).

A possible explanation for these relations is the influence of ID on the O_2_ cascade. The Fick principle states that V̇O_2_ is the product of maximal cardiac output and arteriovenous oxygen extraction (Ca-vO_2_). Both were impaired in patients with ID in a study combining CPET with echocardiography (Martens et al., 2021). Ca-vO_2_ is determined by lung O_2_ diffusion, peripheral oxygen extraction, and O_2_ carrying capacity of Hb (Poole et al., 2018). Iron is also a crucial cofactor in mitochondrial oxidative phosphorylation (Gevaert et al., 2019). Thus, ID can reduce cardiac output through impaired mitochondrial function in cardiac myocytes, as well as Ca-vO_2_ through reduced O_2_ carrying capacity of Hb and impaired skeletal myocyte mitochondrial function.

This is the first study to demonstrate a significant association between iron parameters and diastolic function. In an observational study including 15 patients with HFpEF and ID, Kasner et al. (2013) could not find a relationship between iron parameters and invasive diastolic function. In our study, the relationship was weak after adjustment for age and sex in a multivariable linear regression model. However, when stratifying the population for the presence of ID, the relationship between diastolic function and iron parameters proved to be stronger in patients with ID.

From these data, it may be hypothesized that in patients with ID, iron depletion directly or indirectly impairs diastolic function. Mechanistic animal studies provide further insight in this relationship. Inducing severe ID-anemia in healthy rats directly caused diastolic dysfunction and HFpEF, associated with cardiomyocyte hypertrophy and fibrosis, as well as lung edema (Naito et al., 2009). In another study of the Dahl salt-sensitive rats, animals fed on high-salt diet rats developed not only HFpEF but also ID and mild anemia (Naito et al., 2011). This was associated with a downregulation of hepcidin, but in sharp contrast to ID without HFpEF induced by iron-depleted chow with normal salt content, this was related to a reduced duodenal iron absorption (Naito et al., 2011). Furthermore, iron-depleted cardiomyocytes have reduced mitochondrial function, causing a reduction in diastolic function (up to 85%) which is more important than the reduction in systolic function (up to 64%) (Hoes et al., 2018). Combined with our observations that ID relates to diastolic function and aerobic exercise capacity in patients with ID, these findings suggest that ID could play a more direct role in modulating diastolic function than previously thought. However, the cross-sectional nature of our study does not allow to separate cause and effect. Longitudinal studies and intervention studies, such as the upcoming effect of IV iron in patients with HFpEF (FAIR-HFpEF) randomized clinical trial, will provide more insight in the putative causal role of ID in HFpEF.

The cluster analysis provided another piece of this puzzle. When machine learning categorized patients with HFpEF in an unbiased way, three phenogroups were identified. The comorbidity profile of two of these phenogroups is familiar to many clinicians. The first can be summarized as *atrial fibrillation* + *chronic kidney disease* (AF-CKD) and the second as *male* + *coronary artery disease* (M-CAD). Similar phenogroups have been identified among other HFpEF populations subjected to machine learning analysis. Kao et al. (2015) first applied clustering in HFpEF using 11 features of patients in two randomized clinical trials. Six subgroups were discovered among which the AF-CKD and M-CAD groups can be recognized from this study. Shah et al. (2015) studied 46 features from a single-center HFpEF population. They could discern three phenogroups, including an AF-CKD population, whereas sex and CAD were more evenly distributed across subgroups. More recently, Hedman et al. (2020) found that mortality differed between six phenogroups in a European HFpEF cohort.

We extended this existing literature by incorporating iron parameters into a clustering analysis for the first time. This resulted in a phenogroup of predominantly female patients, who had a comparatively low prevalence of traditional HFpEF risk factors, such as advanced age, hypertension, obesity, CAD, and atrial fibrillation (phenogroup 1). These patients did have a remarkably high prevalence of ID of 86% (*p* < 0.001 vs. other phenogroups). Of note, Kao et al. (2015) and Shah et al. (2015) previously found phenogroups with comparable low-risk profiles, but could not link this to ID as iron status was not determined.

This *low risk* + *iron deficiency* (LR-ID) phenogroup shows a significantly higher peak V̇O_2_ than the other phenogroups, which seems to contradict the relationship of ID with worse peak V̇O_2_. However, patients in the LR-ID phenogroup were also younger, and the percentage of predicted peak V̇O_2_ (more suitable to compare patients of different age distribution) was similar to other phenogroups.

Our findings have important therapeutic implications, as ID is readily treatable by oral or intravenous supplementation (Jankowska et al., 2013). In patients with HFrEF, the latter is preferred after randomized trials demonstrated that intravenous ferric carboxymaltose improved aerobic exercise capacity, symptoms, and quality of life in patients with HFrEF, whereas oral iron failed to improve symptoms (Anker et al., 2009; Ponikowski et al., 2015, 2020; Lewis et al., 2017). Mechanistic studies showed that in HFrEF, ID manifests as both myocardial and skeletal muscle iron depletion; however, only myocardial iron was repleted by intravenous iron (Melenovsky et al., 2018; Núñez et al., 2020). Hopes are high that future studies, such as the ongoing FAIR-HFpEF trial, will demonstrate similar effects of iron administration in HFpEF.

Our study has some limitations. The observational nature of the study precludes definite conclusions about causation. OptimEx-Clin was not powered for clinical outcomes, and together with the cross-sectional design of our study, this did not allow a prognostic assessment based on phenogroup membership. The phenogroup analysis should be validated in an external population before the clinical application of our findings. Finally, the exercise intervention in OptimEx-Clin may have induced a selection bias toward less symptomatic patients with HFpEF, filtering out those who are unable to exercise.

## Conclusion

ID is a frequent and important comorbidity in patients with HFpEF. Lower iron parameters are associated with worse diastolic function and worse aerobic exercise capacity but only in patients with ID. Patient phenotyping by means of machine learning is significantly impacted by including ID. We identified a phenogroup of younger female patients with HFpEF with a high prevalence of ID and a few other HFpEF risk factors. Therefore, we suggest that iron status is routinely checked in all patients with HFpEF, as those with ID should be counseled on possible treatment options.

## Data Availability Statement

The raw data supporting the conclusions of this article will be made available by the authors, without undue reservation.

## Ethics Statement

The studies involving human participants were reviewed and approved by Ethisch Comité UZA, Antwerp University Hospital. The patients/participants provided their written informed consent to participate in this study.

## Author Contributions

AG, UW, BP, VA, MH, and EV contributed to the conception and design of the work. AG, SM, EW, AD, CV, EP-K, PB, and FE contributed to the acquisition, analysis, and interpretation of data for the work. AG drafted the manuscript. All authors critically revised the manuscript, gave final approval and agreed to be accountable for all aspects of work ensuring integrity and accuracy.

## Conflict of Interest

AG reports travel and accommodation funding by Vifor Pharma. EW reported receiving personal fees from Novartis (honoraria for lectures and advisory board activities), Boehringer Ingelheim (honoraria for advisory board activities), and CVRX (honoraria for lectures) outside the submitted work. AD reported receiving grants from Novartis outside the submitted work. CV reported receiving personal fees from Abbott, Daiichi-Sankyo, Bayer and Edwards Lifesciences (lectures) outside the submitted work. BP reported receiving personal fees from Bayer Healthcare (steering committee, lectures), Merck (steering committee, lectures), Novartis (steering committee, lectures), Servier, AstraZeneca (lectures), Bristol-Myers Squibb (lectures), and Medscape (lectures) outside the submitted work. MH reported receiving grants from Novartis (principal investigator of the Activity Study in HFrEF) and personal fees from Bristol-Myers Squibb, Berlin Chemie-Menarini, Novartis, Daiichi-Sankyo, AstraZeneca, Roche, Abbott (advisory board on exercise and diabetes), Sanofi, Pfizer, Boehringer Ingelheim, and Bayer outside the submitted work. EV was supported by an investigator-initiated grant from Vifor Pharma for this work. No other disclosures were reported. The remaining authors declare that the research was conducted in the absence of any commercial or financial relationships that could be construed as a potential conflict of interest.

## Publisher’s Note

All claims expressed in this article are solely those of the authors and do not necessarily represent those of their affiliated organizations, or those of the publisher, the editors and the reviewers. Any product that may be evaluated in this article, or claim that may be made by its manufacturer, is not guaranteed or endorsed by the publisher.
