# Supplementary material for: Iron Deficiency Impacts Diastolic Function, Aerobic Exercise Capacity, and Patient Phenotyping in Heart Failure With Preserved Ejection Fraction: A Subanalysis of the OptimEx-Clin Study
Source: Front Physiol. 2022 Feb 10;12:757268. doi: 10.3389/fphys.2021.757268 (PMC8866976; doi:10.3389/fphys.2021.757268)

Supplemental Materials

Supplemental Table 1: Variables included in the cluster analysis (n=92)...................................... 2

Supplemental Table 2: Characteristics of the study population, stratified according to phenogroup obtained by cluster analysis not including iron deficiency.. 4

Supplemental Figure 1: Gap statistic for cluster analysis, including iron deficiency. 8

Supplemental Figure 2: Gap statistic for cluster analysis, not including iron deficiency................. 9

Supplemental Figure 3: Phenogroups of patients with HFpEF identified through machine learning, and their characteristics, not including iron deficiency... 10

Supplemental Table 1: Variables included in the cluster analysis (n=92).

Category	Variables
Demographics	Age*, Sex*
Past medical history	Atrial fibrillation*, Cardioversion, Cerebrovascular disease*, Chronic kidney disease, Chronic obstructive pulmonary disease, Coronary artery disease*, Coronary artery bypass grafting, Diabetes mellitus*, Hypertension*, Hyperlipidaemia*, Family history of cardiovascular disease*, Myocardial infarction, Percutaneous coronary revascularization, Peripheral vascular disease, Pulmonary vein isolation, Sleep disordered breathing*, Smoking*, Valve surgery, Valvular heart disease
Current medication	Aldosterone antagonist*, Anti-arrhythmic, Anti-diabetic (insulin or oral), Anti-platelet (acetylsalicic acid or other), Beta blocker*, Calcium antagonist*, Diuretic*, Lipid-lowering (statin or other), Oral anti-coagulant (direct or vitamin K antagonist), Renin-angiotensin blocker (Angiotensin conversion enzyme inhibitor or angiotensin receptor blocker)*,
Clinical examination	BMI*, Body height, Body weight, Diastolic blood pressure, Fatigue, Hip circumference, Lung crepitations, Nocturnal cough, NYHA functional class*, Orthopnoea, Peripheral oedema, Raised jugular venous pressure, Rest heart rate*, Systolic blood pressure*, Waist circumference, Waist-hip ratio
Questionnaire	KCCQ physical limitation score, KCCQ symptom stability score, KCCQ symptom frequency score, KCCQ symptom burden score, KCCQ total symptom score*, KCCQ self efficacy score, KCCQ quality of life score,

	KCCQ social limitation score, KCCQ overall summary score, KCCQ clinical summary score
Laboratory analysis	Anaemia, Creatinine, Estimated glomerular filtration rate*, Ferritin, Haemoglobin*, Iron, Iron deficiency*, NT-proBNP*, Total iron binding capacity, Transferrin saturation
Cardiopulmonary exercise test	Peak heart rate, Peak $\dot{V}O_2$*, Peak workload, Percent predicted peak $\dot{V}O_2$, $\dot{V}E/\dot{V}CO_2$ slope*, $\dot{V}O_2$ at aerobic threshold
Echocardiography	A wave, E wave, E' wave (septal), E' wave (lateral), E' wave (average), E/A ratio*, E/e' ratio (septal)*, E/e' ratio (lateral), E/e' ratio (average), Interventricular septum diameter, Left atrial volume index*, LV ejection fraction*, LV end-diastolic diameter, LV end-systolic diameter, LV mass index, LV posterior wall diameter, Right ventricular fractional area change, Tricuspid annular plane systolic excursion*, Tricuspid regurgitation velocity*

All available variables were screened for inclusion. We filtered variables that had >90% identical values, >30% missing values, or correlated at a Spearman correlation coefficient >0.5, keeping the variable that was most informative and had the least missingness. Also, variables that were used in the calculation of another variable were filtered regardless of their correlation coefficient (e.g. weight and height were filtered, and body mass index (BMI) was retained) [16,22]. Thus, we retained 33 of 92 variables. BMI = Body mass index, KCCQ = Kansas City Cardiomyopathy Questionnaire, LV = left ventricular, NT-proBNP = N-terminal pro B-type natriuretic peptide, NYHA = New York Heart Association, $\dot{V}E$ = ventilation, $\dot{V}CO_2$ = carbon dioxide production, $\dot{V}O_2$ = oxygen uptake, * variable included in the final analysis (n=33)

Supplemental Table 2: Characteristics of the study population, stratified according to phenogroup obtained by cluster analysis not including iron deficiency

Characteristic	Phenogroup 1 (n=29)	Phenogroup 2 (n=13)	Phenogroup 3 (n=28)	Phenogroup 4 (n=16)	Phenogroup 5 (n=14)	Phenogroup 6 (n=37)	Phenogroup 7 (n=23)	Phenogroup 8 (n=16)	P value
Age (years)	66.0 (61.0, 72.0)	65.0 (52.0, 69.0)	76.0 (72.8, 78.0)	74.5 (65.8, 76.0)	72.0 (66.2, 74.8)	70.0 (68.0, 72.0)	68.0 (63.5, 71.5)	78.0 (74.8, 81.2)	<0.001
Sex (n, % female)	28 (97)	3 (23)	0 (0)	1 (6)	13 (93)	34 (92)	22 (96)	100 (100)	<0.001
**Past medical history**									
Atrial fibrillation	3 (10)	0 (0)	15 (54)	0 (0)	0 (0)	11 (30)	5 (22)	15 (94)	<0.001
Cerebrovascular disease	0 (0)	0 (0)	2 (7)	5 (31)	1 (7)	6 (16)	1 (4)	5 (31)	0.004
Chronic kidney disease	8 (28)	1 (8)	14 (50)	2 (12)	5 (36)	13 (35)	10 (43)	11 (69)	0.007
Chronic obstructive pulmonary disease	0 (0)	0 (0)	7 (25)	0 (0)	1 (7)	3 (8)	2 (9)	0 (0)	0.009
Coronary heart disease	0 (0)	0 (0)	17 (63)	16 (100)	0 (0)	0 (0)	12 (52)	5 (36)	<0.001
Diabetes	3 (11)	0 (0)	16 (57)	8 (50)	5 (36)	8 (22)	1 (4)	5 (31)	<0.001
Family history of cardiovascular disease	4 (14)	5 (42)	5 (18)	1 (6)	3 (23)	5 (14)	14 (61)	2 (12)	<0.001
Hypertension	8 (28)	11 (85)	28 (100)	14 (88)	14 (100)	37 (100)	22 (96)	16 (100)	<0.001
Hyperlipidaemia	15 (52)	4 (42)	23 (82)	16 (100)	10 (71)	18 (49)	21 (91)	15 (100)	<0.001
Peripheral vascular disease	0 (0)	1 (8)	3 (11)	2 (12)	0 (0)	1 (3)	2 (9)	0 (0)	0.331
Sleep apnoea	2 (7)	0 (0)	20 (71)	2 (13)	2 (15)	3 (8)	4 (17)	0 (0)	<0.001
Smoking, current or previous	14 (48)	7 (54)	23 (82)	9 (56)	3 (21)	6 (16)	14 (61)	3 (19)	<0.001
Valvular heart disease	2 (7)	0 (0)	3 (11)	1 (6)	0 (0)	0 (0)	1 (4)	2 (12)	0.396
**Medication use**									

ACE inhibitor or ARB	0 (0)	8 (62)	24 (86)	15 (94)	14 (100)	36 (97)	19 (83)	13 (81)	<0.001
Aldosterone antagonist	0 (0)	0 (0)	7 (25)	0 (0)	0 (0)	6 (16)	3 (13)	3 (19)	0.018
Anticoagulant	4 (14)	0 (0)	15 (54)	2 (12)	3 (21)	11 (30)	6 (26)	15 (94)	<0.001
Antiplatelet	5 (17)	3 (23)	16 (57)	14 (88)	2 (14)	8 (22)	13 (57)	0 (0)	<0.001
Beta blocker	7 (24)	3 (23)	21 (75)	12 (75)	2 (14)	32 (86)	22 (96)	15 (94)	<0.001
Calcium antagonist	1 (3)	5 (38)	14 (50)	2 (12)	2 (14)	23 (62)	6 (26)	10 (62)	<0.001
Diuretic	4 (14)	0 (0)	25 (89)	5 (31)	10 (71)	27 (73)	19 (83)	12 (75)	<0.001
Glucose lowering	2 (7)	0 (0)	14 (50)	8 (50)	3 (21)	6 (16)	1 (4)	5 (31)	<0.001
Lipid lowering	4 (14)	4 (31)	22 (79)	14 (88)	6 (43)	15 (41)	17 (74)	14 (88)	<0.001
**Clinical examination**									
Blood pressure, systolic (mmHg)	125.0 (120.0, 130.0	125.0 (122.0, 140.0)	130.0 (120.0, 140.0)	130.0 (118.8, 138.8)	135.0 (120.0, 140.0)	130.0 (120.0, 135.0)	125.0 (113.5, 130.5)	132.0 (125.0, 138.5)	0.426
Blood pressure, diastolic (mmHg)	80.0 (70.0, 80.0)	80.0 (74.0, 85.0)	70.0 (64.5, 78.5)	70.5 (70.0, 75.0)	75.0 (73.5, 80.0)	76.0 (67.0, 80.0	80.0 (67.0, 80.0)	76.0 (65.0, 80.0)	0.471
Body mass index (kg/m^2)	28.7 (24.2, 31.2	27.4 (25.5, 29.3)	31.8 (28.2, 34.0)	27.3 (26.5, 28.5)	29.9 (27.4, 34.0)	29.7 (27.1, 32.8)	28.1 (25.5, 31.8)	32.3 (29.8, 34.6)	0.025
KCCQ symptom score	67.0 (46.0, 75.0	68.5 (60.5, 75.2)	73.5 (61.0, 85.8)	79.5 (74.5, 84.2)	65.0 (42.0, 70.0)	73.0 (50.0, 80.0)	69.5 (54.2, 82.2)	73.0 (61.5, 80.8)	0.415
NYHA class<br>II (n,%)<br>III (n,%)	 25 (86)<br>4 (14)	 12 (92)<br>1 (8)	 15 (54)<br>14 (46)	 16 (100)<br>0 (0)	 6 (43)<br>8 (57)	 35 (95)<br>2 (5)	 20 (87)<br>3 (13)	 1 (6)<br>15 (94)	 <0.001
Rest heart rate (bpm)	68.0 (63.0, 74.0)	62.0 (56.0, 74.0)	65.0 (59.5, 73.0)	58.0 (55.0, 70.0)	68.5 (60.8, 76.5)	61.0 (54.0, 66.0)	63.0 (57.0, 78.0)	64.5 (57.8, 68.2)	0.011
**Laboratory analysis**									
Iron deficiency (n, %)	21 (84)	9 (62)	10 (37)	7 (44)	9 (75)	14 (41)	19 (83)	12 (75)	<0.001
Iron (μmol/L)	94.0 (72.0, 112.0)	99.0 (73.8, 114.0)	84.0 (71.0, 116.2)	98.5 (88.2, 112.2)	92.3 (74.0, 120.6)	97.3 (79.9, 114.0)	85.0 (66.5, 102.8)	87.2 (72.8, 99.0)	0.846
Ferritin (μg/L)	58.7 (33.6, 91.3)	86.0 (66.6, 145.0)	142.0 (63.0, 271.9)	123.7 (51.4, 148.9)	88.6 (74.0, 153.2)	115.0 (53.0, 250.0)	62.3 (42.3, 96.0)	63.5 (41.0, 118.0)	0.026

Transferrin saturation (%)	27.8 (20.6, 31.8)	28.6 (21.1, 32.0)	24.1 (21.1, 31.4)	27.3 (23.6, 34.3)	26.7 (18.8, 38.7)	27.8 (23.5, 31.9)	24.1 (17.3, 28.8)	22.8 (19.1, 25.9)	0.540
Anaemia (n, %)	4 (15)	1 (8)	14 (50)	1 (6)	2 (15)	5 (14)	6 (26)	3 (19)	0.005
Haemoglobin (g/L)	13.7 (13.0-14.1)	14.8 (14.1, 15.4)	12.9 (11.6, 14.4)	14.6 (13.4, 15.6)	13.3 (13.0, 14.2)	13.4 (12.9, 14.4)	13.2 (12.3, 13.9)	13.6 (12.5, 13.9)	0.005
EGFR (ml/min/1.73m^2)	86.2 (74.2 , 95.6)	89.0 (81.3, 92.3)	54.9 (37.8, 76.2)	70.9 (65.2, 90.6)	72.3 (62.9, 80.0)	73.7 (59.8, 85.9)	63.1 (51.5, 72.5)	59.3 (55.9, 68.6)	<0.001
NT-proBNP (pg/mL)	155.0 (63.8, 245.2)	72.0 (51.0, 203.0)	607.0 (382.5, 1055.8)	182.0 (67.5, 385.0)	217.5 (129.0, 308.0)	299.0 (122.0, 590.0)	388.0 (240.0, 671.5)	730.0 (363.0, 1261.2)	0.024
**Cardiopulmonary exercise test**									
Peak heart rate (bpm)	144.5 (122.9, 158.0)	150.0 (124.0, 171.0)	106.5 (95.8, 133.0)	117.6 (103.4, 130.9)	129.7 (119.1, 136.8)	117.5 (101.1, 134.4)	115.9 (107.1, 129.3)	103.7 (95.9, 123.3)	<0.001
Peak VO_2 (mL/kg/min)	19.4 (17.6-23.2)	25.8 (21.4, 27.2)	16.5 (13.3, 19.9)	23.0 (17.8, 25.1)	17.4 (14.3, 19.6)	18.4 (16.6, 21.3)	17.8 (13.5, 20.9)	13.5 (11.9, 16.9)	<0.001
Peak workload (W)	107.0 (82.0, 123.3)	140.0 (122.0, 170.0)	92.3 (75.0, 112.9)	125.8 (107.0, 170.5)	92.5 (75.8, 101.3)	93.3 (81.7, 112.0)	80.0 (67.5, 109.0)	64.2 (61.3, 70.4)	<0.001
Percent predicted peak VO_2 (%)	82.3 (69.9, 96.7)	96.9 (87.7, 117.7)	79.6 (69.1, 105.1)	109.8 (84.0, 124.3)	78.3 (71.6, 90.4)	81.6 (69.5, 95.7)	80.9 (56.1, 97.8)	71.0 (59.5, 93.6)	0.001
VE/VCO_2 slope	31.1 (28.6, 35.6)	31.0 (27.0, 33.3)	37.8 (30.1, 44.7)	33.4 (32.2, 37.0)	33.8 (29.7, 35.3)	29.9 (26.9, 34.6)	35.4 (31.8, 40.9)	33.7 (31.6, 39.4)	0.001
**Echocardiography**									
E/A ratio	1.0 (0.8, 1.2)	1.1 (0.9, 1.2)	1.1 (0.8, 1.2)	1.0 (0.9, 1.2)	0.8 (0.8, 0.9)	1.2 (0.8, 1.6)	1.1 (0.9, 1.4)	1.4 (0.9, 1.7)	0.422
E/e' ratio, septal	14.5 (13.9, 16.1)	15.2 (12.7, 16.0)	16.0 (13.5, 18.6)	17.3 (14.9, 20.1)	15.0 (13.1, 16.2)	16.0 (12.1, 19.4)	14.7 (13.1, 17.0)	13.2 (11.2, 17.0)	0.113
Left atrial volume index (ml/m^2)	47.6 (41.7, 51.7)	28.7 (27.1, 31.6)	38.9 (33.4, 46.4)	35.0 (32.2, 40.3)	32.4 (28.6, 38.3)	36.2 (31.1, 46.5)	32.3 (26.6, 45.4)	44.2 (41.3, 53.0)	0.002
LV mass index (g/m^2)	145.2 (130.1, 173.8)	143.9 (123.9, 186.5)	265.0 (234.0, 267.4)	193.8 (179.4, 235.8)	176.6 (157.6, 207.4)	164.6 (152.4, 192.8)	136.4 (128.8, 182.0)	200.4 (174.8, 214.8)	<0.001
LV ejection fraction (%)	60.0 (58.0, 62.0)	61.0 (57.0, 69.0)	57.5 (54.8, 61.2)	55.5 (53.8, 59.0)	64.5 (63.0, 67.2)	62.0 (58.0, 64.0)	60.0 (55.5, 64.5)	57.0 (55.0, 59.2)	0.005

PAPs (mmHg)	28.1 (25.8, 31.4	29.4 (24.4, 30.6)	33.9 (27.8, 41.0)	31.0 (28.9, 33.6)	30.8 (29.9, 34.2)	28.4 (23.9, 33.7)	30.6 (26.6, 33.7)	30.9 (26.0, 35.4)	0.417
TAPSE (mm)	21.5 (18.7, 24.4	22.2 (21.2, 23.8)	20.1 (18.1, 23.8)	22.9 (20.4, 25.1)	22.5 (20.6, 23.4)	22.5 (20.6, 24.1)	20.5 (18.9, 23.6)	17.8 (14.7, 21.0)	0.037

Variables presented as median (interquartile range), p value from Kruskal-Wallis test. Categorical variables: n (%), Pearson's chi square test. ACE = angiotensin conversion enzyme, ARB = angiotensin receptor blocker, EGFR = estimated glomerular filtration rate, LV = left ventricular, NT-proBNP = N-terminal pro B-type natriuretic peptide, NYHA = New York Heart Association, PAPs = systolic pulmonary artery pressure, TAPSE = tricuspid annular plane systolic excursion, VCO_2 = carbon dioxide production, VE = ventilation, VO_2 = oxygen uptake.

Supplemental Figure 1: Gap statistic for cluster analysis, including iron deficiency.

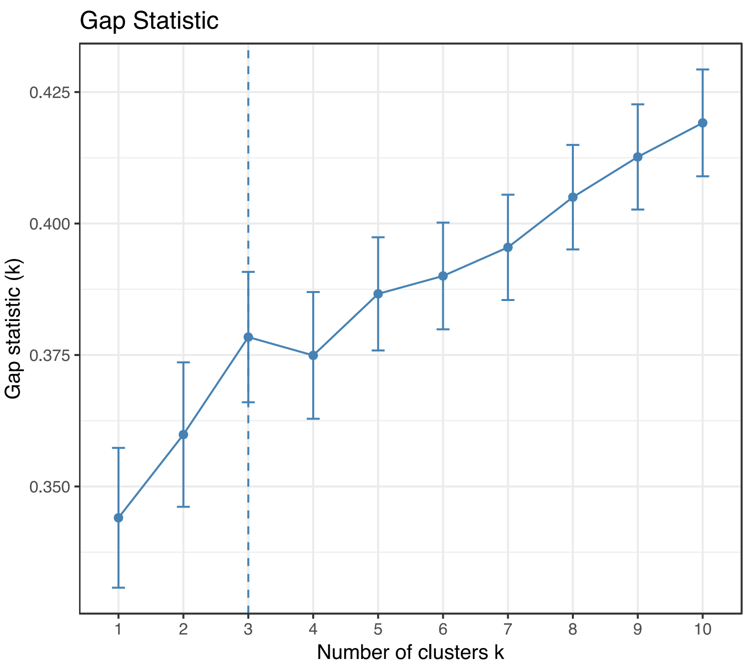

Visualization of the gap statistic, comparing the change in within-cluster dispersion with that expected under a null distribution [25]. In this case, indicating k=3 clusters is the optimal number of phenogroups in our dataset, including iron deficiency as variable.

Supplemental Figure 2: Gap statistic for cluster analysis, not including iron deficiency.

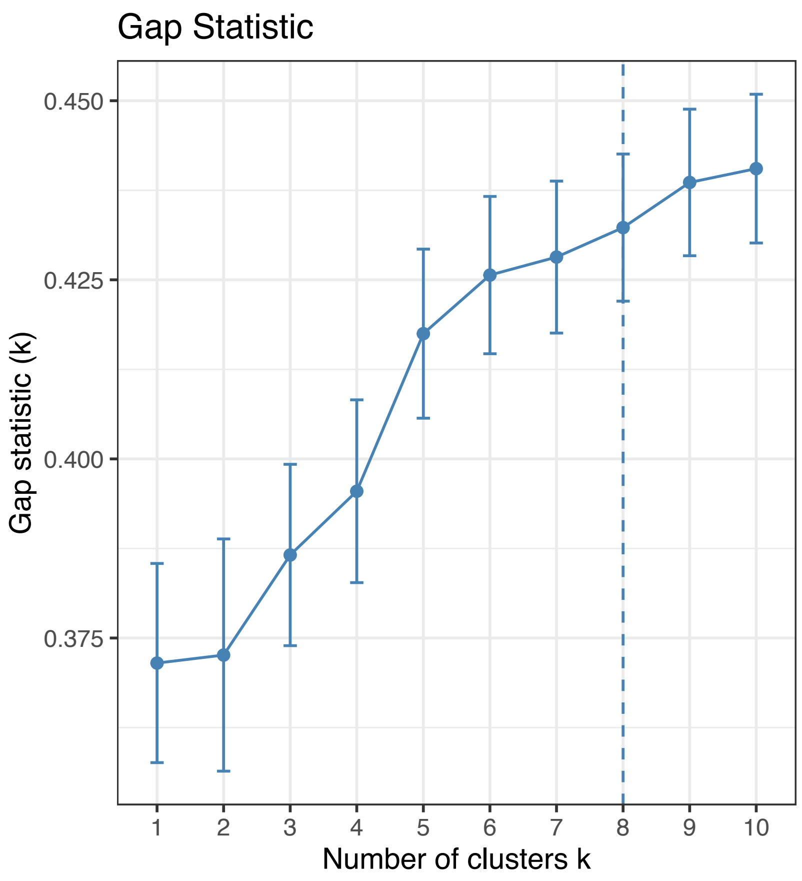

Visualization of the gap statistic, comparing the change in within-cluster dispersion with that expected under a null distribution [25]. In this case, indicating k=8 clusters is the optimal number of phenogroups in our dataset, NOT including iron deficiency as variable.

Supplemental Figure 3: Phenogroups of patients with HFpEF identified through machine learning, and their characteristics, not including iron deficiency

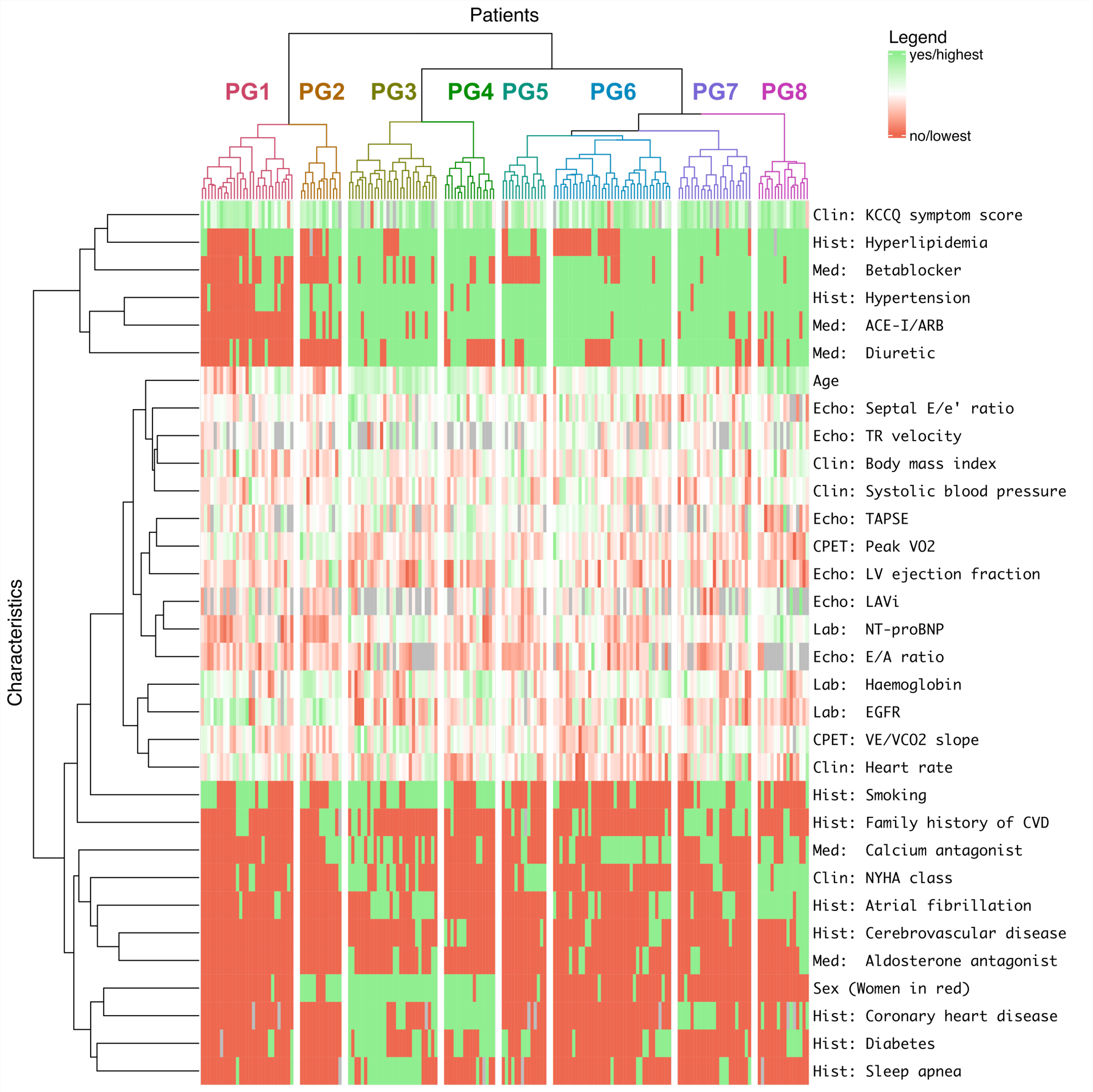

Supplement: Supplementary file 1 [file Data_Sheet_1.PDF]
